# Religiosity prevalence and its association with depression and anxiety symptoms among Hispanic/Latino adults

**DOI:** 10.1371/journal.pone.0185661

**Published:** 2018-02-07

**Authors:** Shir Lerman, Molly Jung, Elva M. Arredondo, Janice M. Barnhart, Jianwen Cai, Sheila F. Castañeda, Martha L. Daviglus, Rebeca A. Espinoza, Aida L. Giachello, Kristine M. Molina, Krista Perreira, Hugo Salgado, Sylvia Wassertheil-Smoller, Robert C. Kaplan

**Affiliations:** 1 University of Massachusetts Medical School, Division of Preventive & Behavioral Medicine, Worcester, MA, United States of America; 2 Albert Einstein College of Medicine, Department of Epidemiology and Population Health, Bronx, NY, United States of America; 3 San Diego State University, Graduate School of Public Health, Division of Health Promotion and Behavioral Science, San Diego, CA, United States of America; 4 University of North Carolina Gillings School of Global Public Health, Department of Biostatistics, Chapel Hill, NC, United States of America; 5 University of Illinois at Chicago, Institute for Minority Health Research, Department of Medicine, Chicago, IL, United States of America; 6 University of Illinois at Chicago, Department of Psychology, Chicago, IL, United States of America; McMaster University, CANADA

## Abstract

**Objectives:**

Religion plays an important role in the lives of people in the United States. We examined the prevalence of religiosity among Hispanic/Latinos in four regions of the United States and looked at its correlation to depression and anxiety symptoms.

**Design:**

The population-based Hispanic Community Health Study/ Study of Latinos enrolled a cohort of Hispanic/Latino adults (*N* = 16,415) ages 18–74 in four US cities from June 2008 to June 2011. Participants with complete data on religiosity (i.e., religious affiliation, frequency of attending religious activities and importance of religion), depression (assessed with the CESD-10), and trait anxiety (assessed with the STAI-10) were included in the present study. Distribution of religiosity is described by sociodemographic characteristics. Associations between religiosity with depression and anxiety were examined with logistic regression models controlling for sex, age group, education, Hispanic/Latino background, clinical center, and nativity.

**Results:**

The majority of the population (89.5%) reported having a religious affiliation. Weekly attendance at religious activities was reported by 41.6% of participants, while 20.6% did not attend any religious activities. Religion was very important to 63.9% and not at all important to 6.7% of the population. The CES-D scores and trait anxiety scores were not significantly related in the overall group to frequency of attending religious activity or perceived importance of religion. However, in age-stratified analyses, among older individuals (65+ years old) reporting “never” participating in religious activities compared to more than once per week was associated with an 80% higher likelihood of having high depressive symptomatology. Similarly, in the older age group, no religious affiliation or reporting that religion is “not at all important” was associated with greater anxiety symptomatology.

**Conclusion:**

Religiosity varied by Hispanic/Latino background. Lack of religiosity was associated with elevated depressive or anxiety symptomology in older adults but not in young or middle-aged adults.

## Introduction

Religiosity is a construct that includes beliefs, practices, and personal devotion relating to religion [1; 2]. An individual's degree of religiosity may be measured by participation in organizational activities such as religious services attendance and in the importance of religion to them [1; 2]. On the whole, 83% of Hispanics/Latinos in the United States embrace a religious affiliation, most often Roman Catholicism; 55% of Hispanics/Latinos are Roman Catholic [3]. Past studies have either focused on Mexican-Americans or have grouped various background subgroups into a single category which ignores the heterogeneity among background subgroups [4].

Depressive and anxiety symptoms have high prevalence in the US Hispanic/Latino population [5–8]. Previous research on participants from the Hispanic Community Health Study/Study of Latinos found that 27% of participants across all Hispanic subgroups reported high levels of depressive symptoms [8]. Factors contributing to increased depressive and anxiety symptoms that tend to disproportionately affect Hispanics include acculturation stress, lack of access to health insurance and medical services, and concerns relating to lack of permanent resident or citizenship status [6; 8–12]. Further, elevated depressive and anxiety symptoms have been well-documented as being associated with chronic disease conditions [8; 13–14] and depression is a risk factor for stroke [15–17].

Prior studies have found that strong religiosity mitigates symptoms of depression and anxiety, by providing a social support system and a stable community of likeminded individuals to serve as a coping mechanism, by providing positive outlooks on stressful situations [18–20]. Religiosity is also associated with reduced mortality [21–22]. For example, Li and colleagues [2016] found that women who attended religious services more than once a week experienced a 33% lower mortality risk. Chida et al. [2009] found that participating in organizational religious activities (e.g., church attendance) increased survival rates in healthy populations. In the present study we assessed the distribution of religiosity measures by various Hispanic/Latino background and socio-demographic subgroups in a cohort of diverse US Hispanics/Latinos adults. Next we assessed the association between religiosity and elevated depressive and anxiety symptoms overall and by age group.

## Methods

### Participants and procedures

This retrospective study analyzes data previously collected for the Hispanic Community Health Study/Study of Latinos (HCHS/SOL). While informed consent was obtained for the larger parent HCHS/SOL study, no interviews were conducted for this smaller sub-study; therefore, no informed consents were separately conducted. The Institutional Review Boards (IRBs) of each respective HCHS/SOL site approved site-specific studies.

Participants in the HCHS/SOL included 16,415 adults aged 18 to 74 years at the time of screening between 2008–2011 who were living in Bronx, NY, Chicago, IL, Miami, FL, and San Diego, CA. Each site was chosen with consideration to the number of Hispanics in each area and to their nations of origin, and each site recruited approximately 4,000 study participants [23]. Persons eligible for the study were community-dwelling men and women who self-identified as being Hispanic or Latino and who were able to travel to a local field center for completion of a study examination. For analyses of Hispanic/Latino group defined by self-reported personal of family national background we defined categories as Dominican, Central American, Cuban, Mexican, Puerto Rican, South American, or Mixed/Other. Individuals who were on active military duty, planning to move away in the next 6 months, and those who were unable to provide consent or to physically come to the clinic were ineligible [24]. Detailed methods of HCHS/SOL have been published elsewhere [23; 24]. Briefly, eligible participants were selected using a probabilistic, two-stage sampling approach. In the first stage of sampling, a stratified-random sample of census block groups was selected within purposively selected US census tracts. Census tracts were chosen to provide diversity within the study population by socioeconomic status, and by Hispanic/Latino group defined by nation of birth or family national background. In the second stage of sampling, households were chosen at random within the randomly-selected census block groups, with over-selection of households that matched with commercially available lists of Hispanic/Latino households. After household rosters were obtained, selection probabilities were assigned to each individual residing in the household in order to produce a final study population in which approximately 6,000 participants were 18 to 44 years of age, and approximately 10,000 were 45 to 74 years of age in each field center. All household members who were selected and who met study inclusion criteria were offered the opportunity to enroll in the study. Of screened individuals who were eligible, 41.7% were enrolled; the remaining screened individuals were either ineligible or refused to participate [23; 24]. All study assessments, including anthropometries, blood draws, and interviews, were conducted by trained clinic staff.

The Albert Einstein College of Medicine’s Institutional Review Board approved this study; the approval number is 2013–2749 and the reference number is 016991. Informed consent was obtained from all participants being included in the original study.

### Measures

Study examinations included completion of standardized clinical measurements and questionnaires administered by a bilingual interviewer in either English or Spanish to obtain baseline information on demographics, smoking behaviors, medical history and other variables.

#### Depressive symptomatology

The presence of depressive symptomatology was assessed using the 10-item Center for Epidemiological Studies Depression Scale (CES-D) [25; 26], which is the shortened version of the original 20-item CES-D scale. The CES-D items ask respondents to endorse statements about how they felt in the past week, such as “I was bothered by things that usually don’t bother me”, “I felt depressed”, and “I was happy”; responses ranged from “rarely or none of the time (<1 day)”, “some or a little of the time (1–2 days)”, “occasionally or a moderate amount of the time (3–4 days)”, or “all of the time (5–7 days”). A cut-point of ≥16 on the complete 20-item CES-D suggests presence of significant high depressive symptoms that was validated using the DSM-III criteria for clinical depression [25; 26].

For the shortened CES-D 10-item scale used in this study, responses were scored with values of 0, 1, 2 or 3, with 0 corresponding to “rarely or none of the time” and 3 to “all of the time”. Positively worded items were reverse coded then the items were summed to create the CES-D10 score with range from 0 to 30, with increasing values indicating higher depressive symptomatology. A cut-point of 10 or higher on the CES-D10 has generally been used for depression screening purposes and has high sensitivity and specificity to the CES-D20 ≥ 16, therefore CES-D10 ≥ 10 was used to define high depressive symptomatology [27]. The Cronbach's alpha for internal consistency for the CES-D10 was 0.82 for the English version and 0.82 for the Spanish version.

Missing any item for the CES-D10 resulted in a missing CES-D10 score. The correlation between the complete 20-item CES-D and the shortened 10-item CES-D was 0.96 [28].

#### State Trait Anxiety

Trait anxiety was assessed by 10 items taken from the State Trait Anxiety Inventory (STAI10) [27], which include questions such as “I feel nervous and restless”, “I feel like a failure”, and “I am a steady person”; responses ranged from “rarely or none of the time (<1 day)”, “some or a little of the time (1–2 days)”, “occasionally or a moderate amount of the time (3–4 days)”, or “all of the time (5–7 days”). Values of the STAI items were assigned values of 1, 2, 3 and 4. Similar to the CES-D10 higher values corresponded to higher anxiety symptomatology and positively worded items were reverse coded. The sum of the 10 items is the STAI10 with range 10–40. High trait anxiety symptomatology was defined as the STAI-10 score greater than or equal to the highest sex-specific quartile (22 for women and 19 for men; hereafter referred to as "trait anxiety"). The Cronbach's alpha for internal consistency for the STAI10 was 0.87 for the English version and 0.85 for the Spanish version.

#### Religiosity measures

The three dimensions of religiosity that were examined, in accordance with the National Institute of Mental Health Collaborative Psychiatric Epidemiology Surveys (http://www.icpsr.umich.edu/icpsrweb/CPES/), are: 1) whether an individual identified with any religion, 2) frequency of attendance at religious services or other types of participation in religious activities, and 3) the importance of religion in the individual’s life. Participants were asked what religion they identified with. Religious affiliation was characterized as a 2-level variable: (1) any religious affiliation or (2) no religious affiliation. To assess frequency of attendance at religious serves participants were asked, “*In general*, *how often do you attend the main worship service of your church or otherwise participate in organizational religion (such as watching services on TV*, *listening to services on the radio*, *participate in Bible study groups*, *etc*.*)*?*”* Response options included “nearly every day,” “at least once a week,” “a few times a year,” “less than once a year,” or “never”. Frequency of participating in religious activities was collapsed into a 3-level category: (1) greater than or equal to once per week, (2) a few times per year, or (3) less than once a year or never.

To assess importance of religion, participants were asked, “*How important would you say that religion and religious beliefs are to you*?*”* Participants responded: "not at all important", "a little important", "somewhat important", "pretty important", "or very important." Finally, we asked participants what religion they identified with.

### Covariate definitions

We identified these variables as potential confounders. We wanted to be as inclusive as possible when running analyses for religiosity and depressive and anxiety symptomology. Age was categorized into 3 groups (18–44, 45–64 and ≥65). Education was categorized as less than high school equivalent, high school equivalent or greater than high school equivalent. Nativity was defined using self-reported data from the baseline examination and categorized as US born if born within 50 US states, Washington, DC, or Puerto Rico, or foreign-born if born outside the 50 US states, Washington, DC, or Puerto Rico. Income was assessed as a 3-level variable (≤$30,000, >$30,000, or missing).

### Statistical analyses

The analytic sample comprised of 15,787 study participants, representing 96.2% of those who were enrolled; 628 participants were excluded because of missing data on depression (n = 411), trait anxiety (n = 140), or religiosity (n = 77). The description of the analytic population is unweighted and describes actual sample sizes; all other estimates in this manuscript, except subgroup n, are weighted to account for the population sampling design. Age- and sex-adjusted distribution of religiosity measures (affiliation, frequency and importance of religion) by sex, age group, Hispanic/Latino background, education, nativity, and interview language were computed using predicted marginal prevalence estimates from weighted logistic regression models. Multivariable analyses were conducted on a subset of participants with complete covariate data; these models were adjusted for age, sex, education, income, Hispanic/Latino background, field center, and nativity. Adjusted mean depression (measured by CESD10) and anxiety (measured by STAI10) scores were calculated using linear regression models. Adjusted prevalence of high depressive and anxiety symptomatology was estimated using predicted marginal from logistic regression models. We calculated adjusted values to account for confounding factors. We tested for statistical interaction between religiosity variables with sex and age group in separate models using interaction terms (i.e. frequency of attending religious activities*sex; frequency of attending religious activities*age group; importance of religion*sex; importance of religion*age group) in adjusted logistic regression models. The tests used are due to the nature of this paper as hypothesis-driven and based on our expertise and review of the literature.

Statistically significant interaction, p<0.05, between religiosity measures and age group was found and thus stratified analyses were done. Rather than p-values, we report confidence intervals. All analyses accounted for complex survey design using SUDAAN 11.0 (RTI, Research Triangle Park, North Carolina) and were weighted to account for the selection of HCHS/ SOL participants with unequal probabilities [24] and were performed using SAS version 9.3 (SAS Institute, Cary, NC) and SUDAAN release 11.0.1 (RTI International, Research Triangle Park, NC).

## Results

Among the 15,787 Hispanic/Latino participants, the mean age was 45.8 (SD = 13.9) years, and 6,319 were male. The study includes Hispanics/Latinos who self-identified as Dominican (1,388), Central American (1,672), Cuban (2,240), Mexican (6,353), Puerto Rican (2,594), South American (1,028), and more than one background or other Hispanic/Latino background (483). Overall, 13,016 of participants were foreign-born; on average, foreign-born participants had resided in the US for 20 (SD 13.9) years. In addition, 12,644 of study participants preferred to be interviewed in Spanish.

### Religiosity

Eighty-nine and a half percent (89.5%) of the HCHS/SOL population identified with a religion. Persons reported identifying as Roman Catholic (58.3%), non-specified Christian (17.2%), Pentecostal (4.5%), Jehovah's Witness (3.0%), Baptist (2.1%), other Protestant (1.1%), Mormon (0.6%), Jewish (0.2%), Muslim (0.03%) or other religion (3.2%). In the HCHS/SOL target population, 41.6% reported attending a religious service at least once a week, 37.9% reported attending religious services a few times a year, and 20.6% reported not attending religious services at all (Table 1). Nearly two-thirds (63.9%) indicated that religion was extremely or very important to them (with 43.6% saying it was extremely important); 21.6% considered religion important, 7.9% said it was somewhat important and only 6.7% indicated that religion was not at all important to them. Due to small percentages in the three middle categories, in subsequent analyses we collapsed the 3 middle categories and report on “extremely important”, “Very important to somewhat important” and “not at all important”. Religious importance varied by Hispanic background, with 51% of Dominicans rating religion as ‘very important’, compared to 33% of Cubans. Cubans were the subgroup least likely to attend religious activities at least once a week (27.4%); comparatively, 48.1% of Central Americans attended religious activities at least once a week.

**Table 1:** Age-and-sex adjusted distribution of frequency of participation in religious activities and importance of religion by socio-demographic characteristics, HCHS/SOL 2008–2010.

		Frequency of Religious Participation			Importance of religion		
		≥ Onetime per week	Few times per year	Never	Extremely important	Very important to Somewhat important	Not at all important
Socio-demographic characteristic	N	% (95% CI)	% (95% CI)	% (95% CI)	% (95% CI)	% (95% CI)	% (95% CI)
Overall*	15,787	41.6 (40.1, 43.1)	37.9 (36.5, 39.2)	20.6 (19.1, 22.1)	43.6 (42.3, 44.9)	49.7 (48.5, 50.9)	6.7 (6.0, 7.5)
Sex ᵃ							
Women	9,468	46.5 (44.6, 48.4)	17.0 (15.5, 18.6)	36.6 (34.9, 38.3)	48.8 (47.0, 50.5)	46.8 (45.2, 48.5)	4.4 (3.7, 5.3)
Men	6,319	36.3 (34.5, 38.0)	24.5 (22.6, 26.5)	39.3 (37.6, 41.0)	38.0 (36.3, 39.7)	52.9 (51.2, 54.6)	9.1 (8.1, 10.2)
Age group ᵇ							
18–44	6,461	35.5 (33.8, 37.3)	22.3 (20.6, 24.2)	42.2 (40.4, 44.0)	38.2 (36.5, 39.9)	54.4 (52.8, 56.0)	7.4 (6.5, 8.5)
45–64	8,060	49.0 (46.8, 51.1)	18.3 (16.5, 20.2)	32.7 (31.2, 34.3)	50.5 (48.6, 52.3)	39.5 (35.3, 43.9)	6.0 (5.0, 7.1)
≥65	1,266	57.1 (53.0, 61.1)	16.6 (13.8, 19.8)	26.3 (22.8, 30.2)	56.5 (52.4, 60.6)	43.6 (41.7, 45.4)	4.0 (2.7, 5.9)
Hispanic Background							
Dominican	1,388	47.6 (43.6, 51.7)	36.4 (32.9, 39.9)	16.0 (13.6, 18.8)	51.0 (47.0, 55.1)	44.6 (40.3, 48.9)	4.4 (2.7, 7.1)
Central American	1,672	48.1 (44.7, 51.5)	36.8 (33.6, 40.2)	15.1 (12.8, 17.7)	56.6 (53.7, 59.4)	38.6 (35.7, 41.5)	4.8 (3.5, 6.6)
Cuban	2,240	27.4 (24.6, 30.4)	32.5 (30.2, 34.9)	40.1 (36.7, 43.6)	33.3 (30.8, 35.9)	54.7 (52.2, 57.1)	11.9 (10.1, 13.9)
Mexican	6,353	47.5 (45.2, 49.7)	41.3 (38.9, 43.6)	11.3 (9.9, 12.9)	43.1 (41.0, 45.1)	52.6 (50.6, 54.6)	4.3 (3.4, 5.5)
Puerto Rican	2,594	38.8 (36.2, 41.4)	35.3 (32.6, 38.2)	25.9 (23.0, 29.0)	45.7 (42.8, 48.7)	46.2 (43.3, 49.1)	8.1 (6.2, 10.6)
South American	1,028	44.8 (40.6, 49.1)	41.3 (37.5, 45.2)	13.9 (11.2, 17.3)	49.8 (45.4, 54.3)	45.8 (41.6, 50.0)	4.5 (3.0, 6.5)
Mixed/Other	483	38.5 (32.9, 44.5)	38.0 (32.0, 44.5)	23.4 (18.1, 29.8)	42.5 (36.2, 49.1)	47.9 (40.9, 55.0)	9.7 (5.6, 16.2)
Education							
Less than HS	5,878	44.0 (41.8, 46.3)	37.5 (35.5, 39.5)	18.5 (16.6, 20.5)	46.8 (44.6, 49.1)	49.1 (47.0, 51.2)	4.1 (3.2, 5.2)
HS equivalent	4,009	40.7 (38.3, 43.1)	39.2 (36.8, 41.6)	20.2 (18.1, 22.4)	42.8 (40.5, 45.1)	50.5 (48.4, 52.7)	6.7 (5.5, 8.1)
Greater than HS	5,583	40.1 (38.2, 42.1)	37.2 (35.2, 39.2)	22.7 (20.7, 24.9)	41.4 (39.5, 43.4)	49.8 (47.9, 51.7)	8.7 (7.5, 10.2)
Income							
≤ $30,000	6,912	42.5 (40.6, 44.5)	36.0 (34.3, 37.8)	21.4 (19.8, 23.2)	45.3 (43.6, 47.0)	48.7 (47.0, 50.5)	6.0 (5.1, 7.1)
> $30,000	7,546	41.2 (39.3, 43.2)	39.8 (37.9, 41.6)	19.0 (17.1, 21.1)	42.2 (40.4, 43.9)	50.6 (48.9, 52.2)	7.3 (6.3, 8.4)
Missing	1,329	38.9 (35.2, 42.7)	35.2 (31.7, 38.9)	25.9 (22.6, 29.4)	43.9 (40.1, 47.6)	50.0 (46.4, 53.6)	6.2 (4.5, 8.4)
Immigration related variables							
Nativity							
US born	2,756	35.8 (33.3, 38.4)	40.1 (37.4, 42.9)	24.1 (21.8, 26.6)	36.6 (34.1, 39.1)	56.0 (53.3, 58.7)	7.6 (6.2, 9.2)
Foreign-born^c^, ≥ 10 yrs	8,707	45.9 (43.7, 48.0)	37.2 (35.3, 39.1)	17.0 (15.2, 18.9)	47.2 (45.4, 49.1)	46.5 (44.7, 48.3)	6.4 (5.4, 7.5)
Foreign-born^c^, < 10 yrs	3,658	38.5 (36.1, 41.0)	37.7 (35.2, 40.2)	23.8 (21.2, 26.7)	43.5 (41.1, 45.9)	49.9 (47.6, 52.1)	6.8 (5.4, 8.4)
Immigrant Generation‡							
First	12,768	43.2 (41.5, 44.9)	37.3 (35.8, 38.8)	19.5 (17.8, 21.4)	45.9 (44.4, 47.5)	47.9 (46.5, 49.4)	6.2 (5.4, 7.1)
Second+	2,984	36.4 (33.8, 39.0)	39.9 (37.3, 42.6)	23.7 (21.5, 26.1)	36.3 (33.9, 38.8)	55.7 (53.1, 58.2)	8.1 (6.7, 9.8)
Age at immigration?							
U.S. Born	2,756	36.5 (33.9, 39.1)	40.4 (37.7, 43.3)	23.1 (20.8, 25.5)	36.7 (34.3, 39.3)	55.9 (53.2, 58.6)	7.5 (6.2, 9.1)
Child (0–12)	1,400	40.9 (36.6, 45.3)	35.5 (31.6, 39.5)	23.7 (18.8, 29.3)	40.8 (37.2, 44.6)	49.9 (46.0, 53.7)	9.3 (6.7, 12.8)
Adolescent (13–19)	2,090	43.5 (40.2, 46.8)	40.2 (37.0, 43.6)	16.3 (14.0, 18.9)	46.0 (42.8, 49.1)	48.8 (45.7, 52.0)	5.2 (4.0, 6.8)
Young Adult (20–44)	7,723	44.8 (42.8, 46.8)	37.0 (35.2, 38.9)	18.2 (16.1, 20.5)	47.0 (45.1, 48.9)	47.1 (45.3, 48.9)	5.9 (4.9, 7.1)
Adult (45–64)	1,667	37.9 (34.3, 41.7)	33.5 (30.2, 37.1)	28.6 (24.7, 32.7)	43.7 (39.6, 47.9)	47.7 (43.5, 52.0)	7.0 (5.1, 9.6)
Older adult (≥65)	151	33.8 (24.3, 44.8)	33.0 (23.0, 44.7)	33.3 (24.0, 44.0)	38.6 (27.3, 51.3)	51.0 (37.6, 64.2)	10.2 (4.4, 21.8)

* unadjusted

ᵃ Adjusted for age group only

ᵇ adjusted for sex only

^c^ Foreign-born refers to person born outside 50 US states or Washington DC

‡ First generation is defined as foreign-born with foreign-born parents

Second+ generation is defined as US-born adults.

All percentages are weighted to account for complex survey design.

Women compared to men were more likely to participate in religious activities at least once per week (46.4% versus 36.3%). Women were also significantly more likely to report that religion is "extremely important" than men (48.8% versus 38.0% respectively). Frequency of participating in religious activities and importance of religion decreased with increasing level of education and income. Foreign-born persons were significantly more likely to report participating in at least one religious activity per week than US born persons (42.6% versus 37.8%).

Table 1 presents the frequency of participating in religious activities increased with age (35.5% in persons age 18–44 vs 57.1% in persons age 65+). Young adults compared to older adults were more likely to report “never” participating in religious activities (42.2% vs 26.3% respectively). We excluded religious affiliation from Table 1 because the vast majority (89.5%) of the analytic population reported some religion.

### High depressive and high trait anxiety symptomatology

Table 2 presents the prevalence of having high depressive and anxiety symptomatology by religiosity measures, after adjustment for sex, age group, education, Hispanic background, clinical center, and nativity. When the entire population was analyzed together, no statistically significant association between high depressive or anxiety symptomatology and religiosity measures was found. Since a statistically significant interaction (P < 0.05) was observed between religiosity variables and age group, analyses were subsequently stratified into three age groups. In a previous paper, we found that there was a higher prevalence of high depressive symptoms in those ages 45–64 than in either the younger or older populations [8].

**Table 2:** Adjusted prevalence of high depressive and anxiety symptomatology by measures of religiosity, HCHS/SOL 2008–2011.

	N	High depressive symptomatology		High anxiety symptomatology	
		Mean (95% CI)	% (95% CI)	Mean (95% CI)	% (95% CI)
Religious affiliation					
Any affiliation	14,557	6.98 (6.84, 7.12)	27.0 (25.8, 28.3)	17.03 (16.88, 17.18)	26.1 (24.8, 27.3)
No affiliation	1,230	6.96 (6.48, 7.43)	27.2 (24.2, 30.5)	17.04 (16.56, 17.53)	25.6 (22.2, 29.2)
Frequency of religious activities					
≥ Once per week	7,519	6.81 (6.60, 7.02)	26.4 (24.8, 28.1)	16.89 (16.69, 17.10)	25.7 (24.0, 27.4)
Few times a year	5,670	7.03 (6.83, 7.24)	27.1 (25.3, 28.8)	17.90 (16.90, 17.28)	26.1 (24.5, 27.7)
Never	2,598	7.22 (6.89, 7.55)	28.5 (26.3, 30.8)	17.21 (16.90, 17.52)	26.5 (23.9, 29.2)
*Test for linear trend*		**0.04**	0.16	0.09	0.61
Importance					
Extremely important	10,538	6.9 (6.5, 7.07)	26.7 (25.1, 28.4)	16.90 (16.70, 17.11)	25.2 (23.6, 27.0)
Very important to Somewhat Important	3,794	7.08 (6.91, 7.24)	27.0 (25.6, 28.4)	17.17 (17.00, 17.33)	26.5 (25.0, 28.0)
Not at all important	768	6.98 (6.34, 7.63)	30.0 (25.2, 35.2)	16.87 (16.27, 17.47)	27.7 (23.1, 32.8)
*Test for linear trend*		0.24	0.32	0.07	0.22

All numbers, except subgroup n, are weighted to account for complex survey design.

High depressive symptomatology was defined as CESD10 ≥ 10.

High trait anxiety stymptomology was defined as STAI10 greater than or equal to sex-specific quartile (22 for women, 19 for men).

All estimates are adjusted for age, sex, education, income, Hispanic background, field center, and nativity.

We examined the association between religiosity (the independent variable) and depression/anxiety (the dependent variables) stratified by age group (Tables 3 and 4). Among those 65 years old or older, never attending religious activities compared to at least once per week was associated with an 80% higher rate of high depressive symptomatology (OR: 1.80 95% CI: 1.11–2.90). Likewise, while high anxiety symptomatology was not associated with religiosity in young adults or middle-aged adults, the 65+ year age group, the highest likelihood of having higher trait anxiety symptoms was observed among those who did not identify a specific religious affiliation and among those who reported that religion was not at all important. No differences in the associations between religiosity variables with high depressive or anxiety symptomatology were observed by sex (data not shown). The presented N refers to the number of participants who identified by the religiosity status.

**Table 3:** Association between high depression symptomatology and measures of religiosity by age group in Hispanic/Latino adults, HCHS/SOL 2008–2011.

	Age Group					
	18–44		45–64		65+	
	N = 6360		N = 7876		N = 1228	
Religiosity	Subgroup N	OR (95% CI) ^a^	Subgroup N	OR (95% CI) ^a^	Subgroup N	OR (95% CI) ^a^
Affiliation						
Any affiliation	5,701	Ref	7,378	Ref	1,170	Ref
No affiliation	657	1.00 (0.77, 1.28)	495	1.09 (0.87, 1.37)	57	0.96 (0.49, 1.87)
Frequency of service						
≥ Once per week	2,434	Ref	4,151	Ref	763	Ref
Few times a year	2,632	1.05 (0.88, 1.26)	2,615	0.96 (0.82, 1.12)	309	1.21 (0.79, 1.84)
Never	1,292	1.05 (0.85, 1.31)	1,107	1.12 (0.88, 1.43)	155	**1.80 (1.11, 2.90)**
*Test for trend*		0.65		0.46		0.03
Importance						
Extremely important	3,775	Ref	5,786	Ref	977	Ref
Very important to Somewhat Important	1,963	1.02 (0.87, 1.19)	1,610	0.98 (0.84, 1.15)	221	1.25 (0.89, 1.75)
Not at all important	393	1.11 (0.79, 1.55)	342	1.33 (0.93, 1.91)	33	1.56 (0.68, 3.58)
*Test for trend*		0.85		0.09		0.62

All numbers, except subgroup n, are weighted to account for complex survey design.

OR (Odds Ratio) describes the odds for having high depressive symptomatology, defined as CESD10 ≥ 10, defined as CESD10 greater than or equal to 10.

ᵃ Adjusted for sex, education, income, Hispanic/Latino background, clinical center, and nativity.

For high depressive symaptomatology, interaction terms with age were not statistically significant for religious affiliation (*P* = 0.58), frequency of religious participation (*P* = 0.12) or importance of religion (*P* = 0.36).

**Table 4:** Association between high trait anxiety symptomatology and measures of religiosity by age group in Hispanic/Latino adults, HCHS/SOL 2008–2011.

	Age Group					
	18–44		45–64		65+	
	N = 6360		N = 7876		N = 1228	
Religiosity	Subgroup N	OR (95% CI) ^a^	Subgroup N	OR (95% CI) ^a^	Subgroup N	OR (95% CI) ^a^
Type						
Any affiliation	5,701	Ref	7,378	Ref	1,170	Ref
No affiliation	657	0.86 (0.67, 1.16)	495	1.08 (0.84, 1.40)	57	**2.08 (1.02, 4.22)**
Frequency of service						
≥ Once per week	2,434	Ref	4,151	Ref	763	Ref
Few times a year	2,632	1.08 (0.91, 1.28)	2,615	0.94 (0.80, 1.10)	309	0.85 (0.55, 1.33)
Never	1,292	0.98 (0.78, 1.24)	1,107	1.21 (0.92, 1.58)	155	1.05 (0.65, 1.68)
*Test for trend*		0.88		0.37		0.98
Importance						
Extremely	3,775	Ref	5,786	Ref	977	Ref
Very important to Somewhat Important	1,963	0.15 (0.97, 1.36)	1,610	0.92 (0.78, 1.08)	221	1.27 (0.85, 1.90)
Not at all important	393	1.02 (0.73, 1.43)	342	1.39 (0.88, 2.18)	33	**2.55 (1.02, 6.38)**
*Test for trend*		0.97		0.34		0.16

All numbers, except subgroup n, are weighted to account for complex survey design.

OR (Odds Ratio) describes the odds for having high trait anxiety symptomatology, defined as STAI10 greater than or equal to sex-specific quartile (22 for women, 19 for men).

ᵃ Adjusted for sex, education, income, Hispanic/Latino background, clinical center, and nativity.

For high trait anxiety symptomatology, significant interactions were observed between age group and for religious affiliation (*P* = 0.0029), frequency of religious participation (*P* = 0.0451) or importance of religion (*P* = 0.14).

## Discussion

This paper adds to the growing literature on the relationship between religiosity and mental health by looking at the relationship in a sample of the U.S. Hispanic community. Religiosity, as indexed by attendance at services and degree of importance of religion and religious beliefs, was highly prevalent in our sample of Hispanic/Latino adults from diverse national backgrounds. The vast majority (90%) reported being affiliated with a religion, 80.4% participating at some level in religious activities in the past year and almost two-thirds (63.9%) reported that religion and religious beliefs are very or extremely important to them. Frequent attendance at a religious service (at least once a week) was most common among Dominicans, Central Americans, South Americans and Mexicans. Religiosity was more prevalent in women than men, and in older age groups than in younger. One possible reason for the lack of difference in the association between religiosity variables with high depressive or anxiety symptomatology, is that women in general tend to report depressive or anxiety symptomology more often than men and thus receive clinical treatment, and as such religiosity might not be solely associated with diminished depressive and anxiety symptomology.

Overall, our data showed no association between religious affiliation, frequency of service attendance, or religious importance with depressive symptomology or trait anxiety. However, in age-stratified analyses, lack of religiosity was moderately associated with elevated depressive and anxiety symptoms in adults age 65 and older, but not in young or middle-aged adults.

Religious affiliation is highly prevalent in Latin America [4] and the majority of our participants were born outside of the mainland United States. Until the 1960’s, at least 90% of Latin America was estimated to identify as Catholic. In more recent decades, across Latin America, there has been a decline in Catholic affiliation, with growing numbers of Protestant or unaffiliated people [3; 4]. Our data thus reflect the variation of religiosity by Hispanic background groups among those who have emigrated from Latin America to the U.S.

Contrary to prior reports, religiosity was not associated with depression or anxiety in our study among young and middle aged-adults. However, among those 65 years old or older, never attending religious activities compared to at least once per week was associated with an 80% increase in high depressive symptomatology. In the same age cohort, the highest likelihood of having higher trait anxiety symptoms was observed among those who did not identify a specific religious affiliation and among those who reported that religion was not at all important. Previous research showing that regular church attendance mitigates depressive and anxiety symptomatology among adults ages 65 and older, regardless of denomination [19; 20; 29]. This could be due to a variety of stressors such as caring for aging parents, distance from families, and lack of financial or emotional support from family members. Attending religious services, in turn, provided a social support network and ameliorated the effects of depressive symptomology [30]. These results also adhere to nationwide data indicating that prayer and attendance at religious services increase in adults 65+ [19; 20; 29]. These results highlight the need for more research on Hispanic older adults with depressive and anxiety symptomatology, particularly in light of the multiple factors impacting depression and anxiety risk in this cohort, such as socioeconomic status, immigration status, and language barriers [3; 20]. The data suggest that the type of social support has changed for the younger age cohorts. In particular, with research suggesting that Hispanics are moving away from Roman Catholicism and towards either Christian evangelicalism or towards religious disaffiliation [3], future studies might look into broader changes in the Hispanic community regarding group cohesion and acculturation. Furthermore, this paper did not address spirituality directly; future research might focus on a trend towards spirituality rather than religious practice.

In the National Institute of Mental Health’s Collaborative Psychiatric Epidemiological Survey (CPES), a multi-ethnic nationally representative study of adults in the US, attending religious activities less than one time per week compared to attending more than one time per week was associated with elevated anxiety symptoms but not depressive symptoms [31]. The discrepancy between studies may be attributed to the differences in the definition of anxiety. Anxiety in the CPES was defined by the Diagnostic and Statistical Manual of Mental Disorders, Fourth Edition (DSM-IV) which includes social phobia, generalized anxiety disorder, post-traumatic stress disorder, and panic disorder. In the present study, anxiety was defined using the State-Trait Anxiety Inventory which may not identify more persons meeting the DSM-IV definition described.

In older adults, more religiosity was associated with less elevated depressive and anxiety symptoms but this was not observed in young or middle-aged adults. Religiosity may help people cope by providing social integration and support. Religiosity may also help by increasing positive emotions, such as optimism, generosity and greater purpose, which can in turn mitigate the symptoms of depression or change the course of depression in the long-term [2; 30].

### Limitations

There are several limitations to the current study. The current study only included religious attendance and importance while excluding other important aspects of religiosity, such as spirituality. This is a cross-sectional study therefore causal inference between religiosity with depression and anxiety cannot be known. Due to the blunt and non-specific nature of the interview questions, participants could have answered in a variety of ways, which limits our interpretation of the data. In particular, both the CESD-10 and the Strait Trait Anxiety-7 have limitations, as they only ask participants how they have felt in the past week, while depression and anxiety respectively are ongoing disorders; therefore, participants might have been misclassified.

Additionally, the results of our study may not be generalizable to other Hispanic populations. For example, SOL was conducted in four urban areas (Chicago, Miami, New York, and San Diego), whereas the results might differ for rural Hispanics who may be more (or less) homogeneously involved in religious activities. The present study describes the distribution of religiosity in a large diverse sample of US Hispanics/Latinos adults. Though the vast majority of the sample identified as being affiliated with a religion, participation in religious activities and importance of religion varied widely by Hispanic background.

Overall, religiosity was not associated with elevated depressive and anxiety symptoms. However, in older adults only, religiosity was modestly associated with less depression and anxiety. Long-term longitudinal studies are needed to further understand how the role of religion changes over a lifetime. Future studies including social support are needed to understand the mechanism between religiosity and depression in Hispanic adults. Furthermore, while the majority of the literature reviewed (including for this paper) highlight the positive associations between religiosity and health, more studies are needed to understand potential associations between religiosity and negative health outcomes among Hispanics, such as refusing medical treatment that conflicts with religious beliefs [32; 33].
